# Bubbles in live-stranded dolphins

**DOI:** 10.1098/rspb.2011.1754

**Published:** 2011-10-12

**Authors:** S. Dennison, M. J. Moore, A. Fahlman, K. Moore, S. Sharp, C. T. Harry, J. Hoppe, M. Niemeyer, B. Lentell, R. S. Wells

**Affiliations:** 1 10 Liberty Way no. 102, 851 Indiana Street no. 307, San Francisco, CA, USA; 2 Department of Biology, Woods Hole Oceanographic Institution, Woods Hole, MA 02543, USA; 3 Department of Life Sciences, Texas A&M University-Corpus Christi, 6300 Ocean Drive, Unit 5892, Corpus Christi, TX 78412, USA; 4 Marine Mammal Rescue and Research, International Fund for Animal Welfare, 290 Summer Street, Yarmouth Port, MA 02675, USA; 5 Chicago Zoological Society, c/o Mote Marine Laboratory, 1600 Ken Thompson Parkway, Sarasota, FL 34236, USA

**Keywords:** stranding, decompression sickness, gas bubbles, diving physiology, marine mammals

## Abstract

Bubbles in supersaturated tissues and blood occur in beaked whales stranded near sonar exercises, and post-mortem in dolphins bycaught at depth and then hauled to the surface. To evaluate live dolphins for bubbles, liver, kidneys, eyes and blubber–muscle interface of live-stranded and capture-release dolphins were scanned with B-mode ultrasound. Gas was identified in kidneys of 21 of 22 live-stranded dolphins and in the hepatic portal vasculature of 2 of 22. Nine then died or were euthanized and bubble presence corroborated by computer tomography and necropsy, 13 were released of which all but two did not re-strand. Bubbles were not detected in 20 live wild dolphins examined during health assessments in shallow water. Off-gassing of supersaturated blood and tissues was the most probable origin for the gas bubbles. In contrast to marine mammals repeatedly diving in the wild, stranded animals are unable to recompress by diving, and thus may retain bubbles. Since the majority of beached dolphins released did not re-strand it also suggests that minor bubble formation is tolerated and will not lead to clinically significant decompression sickness.

## 1. Introduction

During exposure to elevated pressure, increased levels of constituent gases, primarily nitrogen, dissolve in the tissues of air-breathing animals. The amount of nitrogen dissolved in the tissues is a function of the pressure and dive duration. The tissue tension of dissolved nitrogen continues to increase until equilibrium with the environment occurs, at which time the organism is said to be saturated, although specific tissue nitrogen values depend on the lipid content and the dive depth and duration [1]. If the hyperbaric exposure is long enough, the ambient pressure will allow nitrogen to equilibrate, then, as pressure decreases with ascent, the partial pressures will exceed the solubility of nitrogen during the decompression phase, a term called super-saturation. It is widely accepted that increased super-saturation leads to the formation of bubbles during decompression given suitable nuclei [2], and that the presence of gas bubbles in tissues or the circulatory system leads to decompression sickness (DCS) either directly by ischaemia or indirectly by triggering an immune response [3–6]. This was first described iatrogenically and therapeutically with ‘bubbled’-oxygenated blood in humans, with complement activation, disseminated intravascular coagulation (DIC), protein denaturation and leucocyte activation [7]. For humans breathing air while diving, the only current method of avoiding DCS is to carefully control the ascent according to published guidelines for dive depth, duration and rate of decompression to avoid critical super-saturation. While some level of super-saturation may be tolerated, current research suggests that any hyperbaric exposure has a finite probability of DCS [8,9] and divers do sometimes express symptoms even when decompression tables have been strictly followed.

DCS is mainly a phenomenon that occurs in terrestrial animals in a simulated chamber and humans subjected to a hyperbaric environment, and it has been assumed that free diving marine mammals do not have similar problems during natural dives. This cannot be explained merely by the absence of lung ventilation under pressure in marine mammals. It has been suggested that respiratory system compression and collapse limits diffusion, and diving induced blood-flow changes may reduce the amount of nitrogen that equilibrates with the tissues [10,11]. Still, marine mammals appear to experience elevated blood and tissue nitrogen levels while diving. Studies on marine mammals have measured arterial nitrogen tensions (PN2) exceeding three atmospheres absolute (ATA) in the seal during a single dive [1,12], and repeated diving has shown muscle PN2 levels to exceed four ATA in the bottlenose dolphin [13]. In addition, a recent study on dead bycaught marine mammals suggested that bubbles form post-mortem after incomplete decompression owing to elevated nitrogen levels following natural dives [14]. Theoretical studies of inert and metabolic gas dynamics in phocids and odontocetes, using a model that was calibrated against actual measured gas levels, show that marine mammals live with extremely high nitrogen levels [15–17] and allometric scaling of risk suggests that these levels would cause greater than 50 per cent DCS risk in land mammals [16]. It has been suggested that a combination of physiological and behavioural responses may reduce DCS risk [10,15,16,18] in marine mammals normally. Necropsy results from recent stranding events with DCS-like symptoms [19] suggested that marine mammals may exceed a critical threshold at which nitrogen levels may form symptomatic bubbles under certain circumstances. While dive data loggers have made it possible to increase our understanding of the natural dive behaviour in many diving species, our understanding of marine mammal diving physiology for most species other than bottlenose dolphins and a few seal species is extremely limited.

Bubble formation is believed to be the crucial event in the aetiology of DCS, and Doppler ultrasound has been used for detection of intravascular bubbles. The correlation between DCS risk and bubble density is ambiguous [20,21], but a relationship between saturation depth and bubble density has been shown in humans [22]. For a single captive bottlenose dolphin trained to undergo 10–12 serial open water dives to depths of 30–100 m, it was not possible to detect bubbles in fast responding compartments [23]. Captive dolphins have not undertaken the sustained routine diving behaviour of wild animals; therefore, the tissue nitrogen distribution after a few open water dives may not be relevant to wild animals [16,17]. Furthermore, bubbles do not develop ubiquitously in supersaturated animals, yet this experiment had a sample size of one, which is hardly sufficient evidence in itself. In addition, nitrogen removal from fast compartments at the beginning of a diving bout returns to ambient levels within approximately 2 min [10,12]. This is similar to the time lag in the study by Houser *et al*. [23] before measurements could be made. In contrast, computer tomography (CT), gross necropsy and histopathology studies of wild marine mammals that accidentally died at depth in fishing nets and were then hauled fresh-dead to the surface have demonstrated extensive multi-focal intravascular and intraparenchymal post-mortem gas bubble formation, suggesting that these seals, dolphins and porpoises died in a supersaturated state [14]. Some of the original studies proposing complete lung collapse note that blood drawn at depth formed bubbles when left at the surface for 4–7 h, inferring that some degree of super-saturation was, in fact, present [1]. These observations provided the motivation for the current study.

We hypothesized that: (i) gas bubbles formed before or during stranding could be recognized using B-mode ultrasound; (ii) free-ranging repetitively diving dolphins can be chronically supersaturated and at risk for gas bubble formation; and (iii) gas bubble formation will occur when normal diving behaviour is disrupted, such as by stranding and handling.

## 2. Material and methods

Intravascular bubbles, such as those that form in DCS, or that are injected as contrast medium, are represented by hyper-echoic foci within the blood when visualized by brightness mode (B-mode) ultrasound [24–26]. Accumulations of multiple bubbles to form a stationary ‘foam’ such as normally occurs in the gastrointestinal tract result in a specific type of reverberation called ring-down artefact. Multiple studies in human divers have used pulse wave or continuous wave Doppler ultrasound of the systemic venous system and B-mode echocardiography since the discovery in the 1960s [27] that it can be used to detect circulating intravascular gas bubbles that form after deep diving.

Ultrasound technique was calibrated in the laboratory using dolphins that had accidentally drowned at depth in regional fisheries (bycaught) and were subsequently shown to have widespread gas bubbling via CT. Two broadband transducers (linear 12 MHz and curvilinear 5 MHz) were used with a portable diagnostic ultrasound unit (Terason 3000, Teratech Corporation, Burlington, MA, USA). The ultrasound settings were optimized for specific tissue evaluation. Frequency/field depth/focal zone for eye was 12 MHz/5 cm/2.8 cm, respectively, and for kidney and liver was 5 MHz/30 cm/13 cm. Scan area, overall gain and frequency were also optimized. The specimens were used to confirm that gas could be identified and to confirm external landmarks for the liver and the kidneys. Maximal depth of penetration for each of the transducers was evaluated so that the correct transducer could be selected prior to beginning the ultrasound examination. To avoid false positives, to be recognized as interstitial or intravascular gas, there had to be ring-down artefact in at least two places within two or more ultrasound scan frames.

Mass- and single-stranded live cetaceans were examined with the same portable ultrasound unit and the eyes, kidneys and right side of the liver were examined for evidence of gas. Additionally, the kidneys of live dolphins captured after net encirclement, support on a stretcher and restraint on the deck of a vessel and then released during health assessments in 2010 in Sarasota Bay, FL, USA were also examined with the same technique [28]. The average water depth of the habitat used by the Sarasota Bay dolphins is about 2 m. An additional case series in 2011 from Sarasota were examined on the left kidney only, using a GE Voluson i portable ultrasound unit with a 2–5 MHz RAB 3D/4D volume transducer (GE Healthcare, Milwaukee, WI, USA). Water and ultrasound gel were used for coupling. Ultrasound examinations preceded any procedures that breached the skin potentially introducing gas iatrogenically, with the exception of the dolphins involved in health assessments that were blood sampled using Vacutainers before removal from the water. In stranded dolphins that either died or were euthanized, ultrasound examinations were followed by whole body CT in some cases and then necropsy examination for correlation of ultrasound findings and to determine the presence of any disease processes, the extent of stranding-related changes and presence of any decomposition. Duplicate tissue samples were collected for histology according to established in-house protocol and submitted for evaluation by board-certified veterinary pathologists with extensive experience in marine mammal pathology. All cadavers were held in a chiller once at the laboratory, with the exception of one animal that had been frozen and then thawed prior to necropsy (CCSN08-206Dd).

## 3. Results

### (a) Validation of ultrasound as a technique for gas bubble detection

Three drowned bycaught dolphins were evaluated in the laboratory using B-mode ultrasound prior to necropsy (table 1). All specimens demonstrated widespread ring-down artefact consistent with the presence of gas bubbles. In one specimen, CT scanning prior to necropsy confirmed the presence of gas prior to disruption of the specimen and demonstrated the extensive distribution of bubbling that involved all abdominal organs, blubber, vasculature and eyes. Gas bubbles were confirmed during necropsy observations. Gross necropsy of all animals and histopathology from two animals confirmed the presence of bubbles and that these dolphins had drowned without evidence of another cause of death. This laboratory-based study confirmed that B-mode ultrasound is capable of detecting gas bubbles in these tissues and species. To ensure that gas bubbles were not found in animals that had died after captivity in a shallow tank, which should preclude significant super-saturation, a harbour porpoise that had been maintained in a tank for six months was examined by CT. No evidence of abnormal gas accumulation was found.

**Table 1.** Summary of cetaceans examined for the presence of bubbles by ultrasound, computer tomography, necropsy and histopathology. Species: La, white sided dolphin (*Lagenorhynchus acutus*); Dd, short beaked common dolphin (*Delphinus delphis*); Tt, bottlenose dolphin (*Tursiops truncatus*). History: B, bycaught; D, stranded dead; E, euthanased; L, ultrasound alive on beach; P, ultrasound post mortem; N, live net restraint, examined on deck and released; R, relocated and released after live stranding; M, mass stranded; S, single stranded; C, CT scanned; X, no sample. Bubbles detected: NO, none seen; LK, left kidney; RK, right kidney; LV, liver; LE, left eye; RE, right eye; MM, muscle; ME, meninges; VC, vena cava; DI, diaphragm; CO, colonic mucosa; LN, lymph node; EC, epicardium; SC, spinal canal; HE, heart; SD, subdermal.

bubbles detected by:									
date	ID	Sp.	total length (cm)	sex	history code	ultrasound	computer tomography	gross necropsy	histopathology
*bycatch*									
5 Feb 2009	DO8481	La	195	M	B	LK RK LE RE MM	LK RK LE RE MM	LK MS RM MM LV PA	X
27 May 2009	DO6689	Dd	135	M	B	LK RK LE RE MM	X	LK RK	LN CO
17 June 2009	DO8155	La	230	M	B	LE MM	X	EC LV LK	LN CO
*mass stranding*									
22 Jan 2009	CCSN08-206	Dd	219	F	M D P	LK RK	ME SC MM LV SD LK RK	NO	X
5 Mar 2010	IFAW10-069	Dd	176	F	M L E C	LK RK	ME MM SC HE SD LK RK	ME LK RK	NO
3 Dec 2009	IFAW09-191	Dd	218	M	M L E	LK RK	MM HE SC LK RK SD	LK RK MM	NO
12 Dec 2009	IFAW09-200	Dd	240	M	M L E	LK RK	X	LK RK VC ME	NO
12 Dec 2009	IFAW09-201	Dd	214	M	M L E	LK RK	X	LK ME DI	DI
26 Mar 2010	IFAW10-113	La	272	M	M L R D	LK RK	X	LK RK	NO
3 Feb 2009	IFAW09-012	Dd	187	M	M L R	LK RK	X	X	X
3 Feb 2009	IFAW09-013	Dd	192	F	M L R	LK RK	X	X	X
3 Feb 2009	IFAW09-014	Dd	183	F	M L R	LK RK	X	X	X
3 Feb 2009	IFAW09-015	Dd	170	F	M L R	LK RK	X	X	X
21 Apr 2009	IFAW09-97	La	267	M	M L R	LK RK LV	X	X	X
3 Dec 2009	IFAW09-192	Dd	210	M	M L R	LK RK	X	X	X
26 Mar 2010	IFAW10-116	La	205	F	M L R	LK RK	X	X	X
26 Mar 2010	IFAW10-114	La	238	F	M L R	LK RK	X	X	X
26 Mar 2010	IFAW10-115	La	251	M	M L R	LK RK	X	X	X
26 Mar 2010	IFAW10-112	La	218	F	M L R	LK RK	X	X	X
12 Mar 2010	IFAW10-091	La	205	F	M L R	LK RK	X	X	X
12 Mar 2010	IFAW10-092	La	213	M	M L R	LK RK	X	X	X
3 Dec 2010	IFAW10-238	Dd	195	F	M L R	LK RK	X	X	X
*single stranding*									
11 Dec 2009	IFAW09-199	Dd	215	M	S R E P	LK RK	X	LK RK	NO
16 Dec 2009	IFAW09-209	Dd	230	M	S D P	LK RK	X	RK	NO
29 Jan 2010	IFAW10-018	Dd	218	M	S L E	LK RK	MM LK RK	LK RK ME	NO
*live restraint*									
18 May 2010	227	Tt	225	F	N	NO	X	X	X
19 May 2010	54	Tt	245	F	N	NO	X	X	X
19 May 2010	229	Tt	180	F	N	NO	X	X	X
19 May 2010	258	Tt	254	M	N	NO	X	X	X
20 May 2010	231	Tt	249	F	N	NO	X	X	X
21 May 2010	211	Tt	231	F	N	NO	X	X	X
16 May 2011	262	Tt	218	M	N	NO	X	X	X
16 May 2011	264	Tt	209	M	N	NO	X	X	X
16 May 2011	266	Tt	214	M	N	NO	X	X	X
17 May 2011	127	Tt	253	F	N	NO	X	X	X
17 May 2011	193	Tt	250	F	N	NO	X	X	X
18 May 2011	268	Tt	266	M	N	NO	X	X	X
18 May 2011	198	Tt	267	M	N	NO	X	X	X
18 May 2011	133	Tt	239	F	N	NO	X	X	X
18 May 2011	10	Tt	259	M	N	NO	X	X	X
19 May 2011	227	Tt	234	F	N	NO	X	X	X
19 May 2011	250	Tt	214	M	N	NO	X	X	X
19 May 2011	90	Tt	259	F	N	NO	X	X	X
19 May 2011	270	Tt	190	M	N	NO	X	X	X
19 May 2011	246	Tt	233	M	N	NO	X	X	X

### (b) Evaluation of cetaceans for gas bubble formation

Twenty-two dolphins that stranded on the beaches of Cape Cod, MA, USA during 2008–2010 were examined for the study (table 1). These included eight *Lagenorhynchus acutus* (Atlantic white-sided dolphin) and 14 *Delphinus delphis* (short-beaked common dolphin). Nineteen dolphins mass-stranded (two or more animals stranding at the same time, excluding mother and calf) in five unrelated events. Three common dolphins stranded individually. Five of the mass-stranded and two of the single-stranded dolphins died or were euthanized on the beach and were not relocated and released. Fourteen mass-stranded dolphins were relocated and released at a site remote from the stranding location. Of those 14 dolphins that were released, two dolphins in two separate mass-strandings were each tagged with a satellite-linked transmitter, and demonstrated normal behavioural patterns post-release. One released mass-stranded dolphin was found re-stranded and dead the following day. The third single-stranded dolphin re-stranded shortly after release and was euthanized that same day.

**Figure 1.**
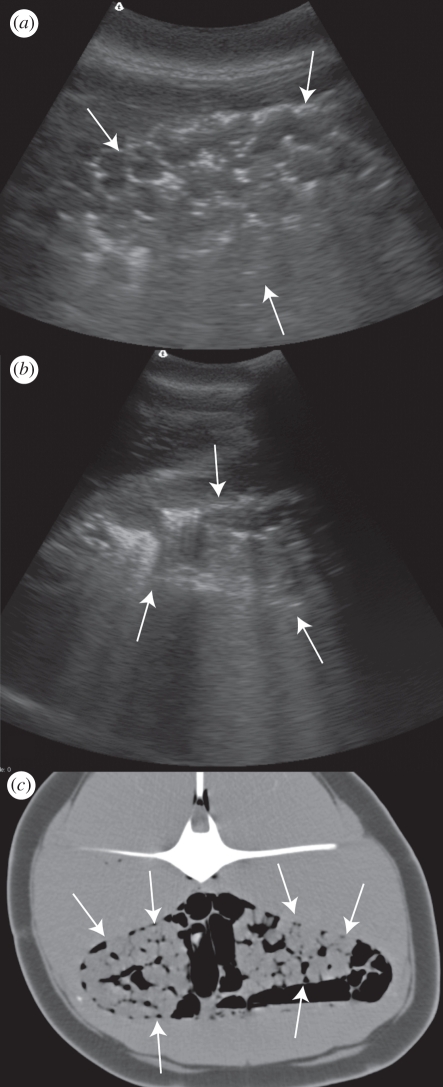
Common dolphin IFAW10-069Dd. Arrows indicate renal margins. (*a*) B-mode ultrasound image of the left kidney showing hyperechogenicities (white) and ring-down artefact around the renules. (*b*) B-mode ultrasound image of the right kidney in the same dolphin with a greater depth of field to enhance the ring-down artefacts. (*c*) Transverse CT image at the level of the kidneys showing gas (black) surrounding and within the kidneys. The animal's left is to the left of the image. Gas is also seen in this image within intestinal loops and adjacent to the spinal cord. The CT was performed within 2 h of death. Window Width (WW) 553, Window Level (WL) 62. Three millimetre slice thickness, soft tissue reconstruction algorithm.

Most dolphins underwent ultrasound examination on the beach prior to any other procedure, and some of those determined fit for release were repeatedly examined via ultrasound during the relocation process. The time between the dolphins physically stranding on the beach and the ultrasound being performed varied from within 2 min for three dolphins that mass-stranded at a location of an ongoing stranding response and 8 h (estimated) for a dolphin found at the high-tide water level early in the morning. In all 22 dolphins, gas bubbles were identified in the region of the kidney bilaterally using ultrasound (figure 1). In 2 of 22 dolphins, gas was concurrently identified in the portal vasculature and of those two dolphins, one was successfully released and one died. Gas was not identified in either eye of any stranded dolphin.

**Figure 2.**
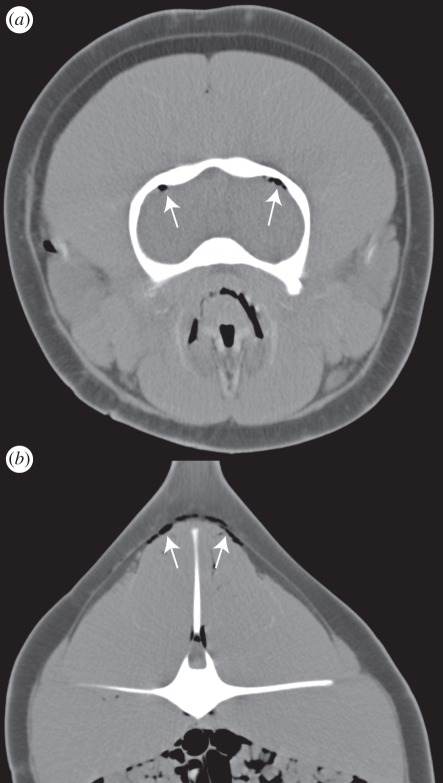
Transverse CT images from the same animal as in figure 1. (*a*) Gas within the subarachnoid space or meningeal vasculature (arrows). (*b*) Gas between the blubber and muscle layers and in linear configurations consistent with intramuscular vasculature (arrows). Intestinal gas is also evident and is normal. Three millimetres slice thickness, soft tissue reconstruction algorithm. (*a*) WW500 WL50; (*b*) WW553 WL62.

Four of nine dolphins that died or were euthanized underwent CT scanning prior to necropsy. In one dolphin, the CT scan took place within 2 h of death and in three dolphins within 12 h of death with the dolphins being kept cool for the time between death and scanning. In all four dolphins, gas-attenuating regions were identified in the region of both kidneys as was identified via ultrasound while the dolphins were still alive (figure 1). There was no CT evidence of gas accumulation in the liver or eyes of these four dolphins, correlating with the ultrasound results for those individuals. On CT examination, all four dolphins had abnormal gas accumulations in locations other than the kidneys. In those dolphins, abnormal gas was identified between the blubber layer and the muscle, in multiple linear accumulations within the musculature that were presumptively intravascular, in the coronary arteries of the heart, within the brain and in the spinal canal (figure 2). It was not always possible to determine whether the gas was intravascular or intraparenchymal (within the tissues) via CT. In addition to abnormal accumulations, normal gas accumulations were identified in the paranasal sinuses, within the upper and lower airways and within the gastrointestinal tract.

Nine of nine dolphins that died or were euthanized underwent careful necropsy evaluation with tissue sampling for histology (with the exception of the animal frozen prior to necropsy). In eight dolphins, gas accumulation in the region of the kidney was confirmed. This was mostly subcapsular but some renal intravascular bubbling was also identified. Gas bubbling at necropsy was often more extensive than had been recognized from CT images owing to the ability to spread out the gastrointestinal tract and its associated mesentery and evaluate the portal and mesenteric vascular system. Histological evidence of intraparenchymal bubble formation was identified in the diaphragm muscle of one live-stranded dolphin.

Long-term resident bottlenose dolphins of the well-studied Sarasota Bay dolphin community [29] were temporarily captured for health assessment [25] after a brief chase, (30 s to several minutes) and encirclement with a seine net in shallow water. They were captured alone, or with one or two other animals and were transferred one by one to the deck of the health assessment vessel within 32–192 min of capture. Blood sampling and other procedures were often performed in the water before the dolphin came on-deck. Ultrasonic evaluation of 20 live bottlenose dolphins 48–210 min after capture and 4–28 min after coming onboard the vessel did not show bubbles in any cases.

## 4. Discussion

B-mode ultrasound consistently detected perirenal bubbles in mass and single-stranded live dolphins. Of the stranded animals, 14 of 22 were translocated and released without obvious detriment. For those animals that died or were euthanized, the presence of bubbles was confirmed in the kidney and other organs by gross necropsy, and in some cases, by CT and/or histology. It is important to discuss the source of these bubbles and their implications for our understanding of how diving mammals sustain extreme dive depth and duration.

### (a) Nature of gas bubbles observed

There are several potential mechanisms that result in accumulation of intravascular and intraparenchymal gas. These include barotrauma (pressure change induced injury to an air-containing structure), gas forming bacteria, absorption of intestinal gas, iatrogenic introduction, decomposition and desaturation or ‘off-gassing’ of supersaturated blood and tissues. The uniformity of the findings of renal gas accumulations in all live-stranded dolphins without adverse clinical consequence in the majority of dolphins is compelling evidence that the aetiology of the gas bubbles was not due to gas-producing bacteria or decomposition. In the dolphins that died or were euthanized, findings were similar and necropsy and histopathology determined that decomposition was not present nor were any signs of systemic infection by gas-producing bacteria. Morbillivirus and influenza can cause necrotizing pneumonia in phocids that can result in alveolar rupture (barotrauma) and mediastinal and interstitial pulmonary emphysema [30,31], however, this has not been described in cetaceans and evidence of pneumonia was not observed. Barotrauma owing to necrosis and vascular compromise of the gastrointestinal tract [32] while stranded on the beach could be an explanation for the portal vein gas identified in two cetaceans, but has previously been explored and rejected as a source for diffuse gas bubbling in stranded cetaceans [33], and would not account for the renal gas observed in these cases. Three dolphins that were examined with ultrasound within 2 min of stranding on the beach already had gas bubble formation in the kidneys, strongly suggesting that this gas was already forming while the dolphins were still in the water (unless they had already stranded and refloated) when disruption to normal behaviour had already begun and before any of the gravitational effects of being out of the water could have occurred. Future studies aimed at gas sampling and analyses to confirm the origin of the gas [34] are needed.

### (b) Super-saturation and gas emboli as part of normal dive cycling

How can marine mammals undertake such extreme depth and duration of dives? The basic assumption has been that there is avoidance of elevated nitrogen pressures that would cause symptomatic bubble formation [35]. Avoidance mechanisms, suggested by computer modelling, include alveolar collapse to minimize diffusion below a certain depth, a slowing of the heart and redistribution of blood to conserve oxygen for vital tissues such as brain [35]. Some [2] have speculated, without convincing others [36], that the physiology and anatomy of dolphins are less generative of bubble nuclei than humans. However, the effect of both the alveolar collapse depth and blood-flow distribution is case specific and may in some cases increase the end-dive blood and tissue PN2 levels [15,18]. Previously, we described drowned bycaught dolphins and seals with post-mortem gas emboli that could indicate routine super-saturation [14]. However, in the discussion of that work, we assumed that the animals drowned at depths below lung collapse, and thus had taken up sufficient nitrogen during their routine dives prior to entanglement to become supersaturated when hauled back to the surface. However, if one assumes that the subject's alveoli were still patent at the depth of entanglement, the nitrogen levels that resulted in super-saturation at the surface could have developed during the terminal struggles prior to death during entanglement, and not reflect the normal dive situation. The potential for deeper depth of lung compression was suggested recently with imaging of cetacean cadavers under various pressures [37]. Thus the bycatch data suggest, but are insufficient to conclude, that dolphins are at risk of bubbling during routine ascents. The propensity to become supersaturated and the consequent development of bubbles depend primarily on the rate and duration of the various components of a dive cycle, including the duration of the surface interval. One way that human divers occasionally treat symptoms of DCS is to return to deeper water with a fresh supply of air. Likewise, dolphins could avoid clinical issues with bubble formation by limiting their time at the surface. It has been suggested that the shallow and short dives that routinely occur in some species between dive bouts serve as recompression dives, and theoretical estimates have shown that this may be an efficient method to combine physiology and behaviour to reduce the risk of bubbles [15,16].

In this light, the act of stranding on a beach with the resultant inability to recompress may be the basis for our ultrasound observations of bubbles. The generally good health of mass-stranded dolphins [38] is in contrast to that of most single-stranded animals, which tend to have a variety of pathological conditions that have driven the animal to beach alone. Single-stranded animals may not have been diving so actively as mass-stranded groups and may have off gassed substantially prior to stranding. Our sample size of single-stranded animals is too small to make any definitive observations, but we would hypothesize that the prevalence of bubbles will be less in most single-stranded than mass-stranded animals, if they are diving less over an extended period. The absence of bubbles in experiments with a diving dolphin [23] likely reflects the fact that this subject was not wild and diving continuously, rather held in shallow water between experimental periods. This dive behaviour will mainly result in nitrogen uptake in fast tissues, e.g. muscle, as previously shown in the same experimental paradigm [13]. The fast removal from such compartments will likely occur within minutes after the animal returns to the surface and thereby remain undetected. Dolphin tissues rank from low- to high-lipid content as follows: muscle, liver/kidney, brain and blubber [39]. The relative infrequency of bubbles detected by ultrasound in the liver when compared with the kidney in this study probably reflects the lesser accessibility to this technique in the liver and portal vasculature, than the more superficial kidneys. The absence of significant bubbles in 20 bottlenose dolphins, restrained on the deck of a health assessment vessel, after swimming wild in an average depth of 2 m of water is commensurate with the minimal risk of super-saturation at such shallow depths.

### (c) Limitations of ultrasound as a bubble detector

The use of ultrasound for the detection of gas in the live dolphin has limitations. Viscera or body systems that normally contain gas, such as the trachea, thorax and gastrointestinal tract, and regions separated from the periphery by gas-filled structures, such as the lung or gastrointestinal tract, cannot be evaluated. Instead, organs that should not contain gas under normal circumstances and lie adjacent to the body wall were considered the best target organs for evaluation in this study. The right aspect of the liver was chosen over the left due to the larger ventral portion that is accessible caudal to the pectoral flipper and because the stomach lies in close association with the left liver lobes. The kidneys are also easily accessible and both liver and kidneys have repeatable locations that can be found using external topographical landmarks of the pectoral flippers and dorsal fin in dolphin species. Cardiac echocardiography is best undertaken with a trans oesophageal probe, in dolphins trained to exhale and hold their breath [40] which is impractical in wild animals. Given our limited ability to detect gas in most organs by ultrasound, much more gas may have been present in the live dolphins that were released as was determined on CT in the dolphins that were euthanized or died. A comparison of the distribution and amount of gas accumulation between those dolphins that died or were euthanized and those that were released could not be performed in this study, but such differences should be evaluated in the future to determine whether or not volume and distribution of gas affects outcome. Ongoing use of CT post-mortem and pre-necropsy will help map gas accumulations in live-stranded dolphins that die or are euthanized and this may locate other areas where gas accumulates repeatedly.

### (d) Significance of bubbles observed

The gas bubbles identified in the region of the kidneys of the dolphins that underwent necropsy were mostly subcapsular and as such considered stationary. It is important to realize there is very little perirenal fat in dolphins. The perirenal bubbles were in loose connective tissue. Currently, any differences between the extent and distribution of gas bubbles in the cetaceans that were successfully released compared with those that died or were euthanized are unknown: both the live-released group and the dead/euthanized group had evidence of renal gas bubbles. In terms of other organs, it is hard to predict the extent of bubbles elsewhere in the body of the animals examined, given that many organs are shielded by normal gas or bone. Given the greater solubility of nitrogen in tissues with high-lipid content [41], and the resultant slower kinetics for bubble formation, the relative distribution of bubbles would be informative. Furthermore, the presence of circulating intravascular gas bubbles is currently unknown in these cases. Circulating gas bubbles may carry a risk of clinically significant DCS via ischaemic damage to organs secondary to capillary or other vessel blockage. Or gas bubbles that are circulating may not increase the risk in vital organs unless there is an arteriovenous shunt, e.g. a patent foramen ovale or a pulmonary shunt, that bypasses the pulmonary capillary bed that acts as a filtration system [42,43]. Studies have shown that DCS is associated with both complement [29,44] and immune activation [4,45]. In addition, stationary intravascular gas microbubbles have been associated with triggering of the complement cascade resulting in thrombus formation and ischaemia occurring secondary to blockage from the thrombi rather than the bubbles themselves [46,47]. Thus, the bubbles may act as foreign bodies that trigger a cascade of events that eventually result in trauma and DCS symptoms. Evaluation for evidence of circulating gas bubbles in addition to stationary accumulations is therefore necessary.

### (e) Future studies

In conclusion, gas bubble formation can occur without clinical consequence in live-stranded dolphins. Repeatedly, this gas is identified in or around the kidneys, however, the extent of gas bubbling is unknown. It is also unclear from our use of B-mode ultrasound, despite multiple time-series samples in some of our cases, if there is a change in bubble density with time since stranding. The reason for this is that the artefact used to detect bubbles is qualitative, not quantitative.

In the absence of data to the contrary, the origin of the gas is most likely desaturation of supersaturated blood and tissues. These results agree with both experimental and theoretical estimates suggesting that marine mammals may live with elevated levels of tissue and blood nitrogen, which in certain circumstances may result in bubbles [12,16,48]. It is unclear if our results reflect the effects of stranding and handling, or if diving mammals are routinely bubbled asymptomatically, in addition to situational clinical bubbling [19]. Ultrasound has primarily been used in dolphins in aquaria [49], and at times in health assessment scenarios for free-ranging dolphins [28,50]. Cardiac ultrasound of live-stranded dolphins is an approach that should be followed in the light of our findings. However, it has been shown that the best echocardiographic images can be obtained using a trans oesophageal probe [40], with the animal trained to exhale and hold its breath. This would not be practical for a stranded case. But transthoracic B-mode imaging should be attempted. This would enable a better understanding of the systemic role of bubbles in stranded animals, but would not address the extent to which super-saturation and bubbles are part of routine diving physiology, as opposed to purely in stranded animals being handled. A pressure proof data-logging ultrasound system attached to a diving cetacean with suction cups could be developed to address this question.
